# Discovery of a Multifunctional Octapeptide from Lingzhi with Antioxidant and Tyrosinase Inhibitory Activity

**DOI:** 10.3390/ph15060684

**Published:** 2022-05-30

**Authors:** Yodying Yingchutrakul, Sucheewin Krobthong, Kiattawee Choowongkomon, Phakorn Papan, Pawitrabhorn Samutrtai, Thanisorn Mahatnirunkul, Thitikorn Chomtong, Nitipol Srimongkolpithak, Theeranuch Jaroenchuensiri, Chanat Aonbangkhen

**Affiliations:** 1 National Omics Center, National Science and Technology Development Agency, Pathum Thani 12120, Thailand; yodying.yin@nstda.or.th; 2 Center for Neuroscience, Faculty of Science, Mahidol University, Bangkok 10400, Thailand; sucheewin82@gmail.com; 3 Interdisciplinary Graduate Program in Genetic Engineering, Kasetsart University, Bangkok 10900, Thailand; kiattawee.c@ku.th; 4 Department of Chemistry, Faculty of Science, Chiang Mai University, Chiang Mai 50200, Thailand; phakorn_papan@cmu.ac.th; 5 Department of Pharmaceutical Sciences, Faculty of Pharmacy, Chiang Mai University, Chiang Mai 50200, Thailand; pawitrabhorn.s@cmu.ac.th; 6 National Nanotechnology Center, National Science and Technology Development Agency, Pathum Thani 12120, Thailand; thanisorn.mah@nanotec.or.th (T.M.); thitikorn.cho@nanotec.or.th (T.C.); 7 National Center for Genetic Engineering and Biotechnology, National Science and Technology Development Agency, Pathum Thani 12120, Thailand; nitipol.sri@biotec.or.th; 8 Center of Excellence in Natural Products Chemistry (CENP), Department of Chemistry, Faculty of Science, Chulalongkorn University, Bangkok 10330, Thailand; pream.theeranuch@gmail.com

**Keywords:** Lingzhi, bioactive peptide, tyrosinase inhibitory peptide, antioxidant peptide, LC-MS/MS, proteomics

## Abstract

*Ganoderma lucidum* or Lingzhi is a fungus species widely known as a traditional medicine. Exploring the beneficial peptides by hydrolysis using pepsin and trypsin has been extensively performed to identify new bioactive natural products. A multifunctional peptide that expresses potential scavenging activity and tyrosinase inhibition is valuable in therapeutic and cosmetic applications. This study aimed to identify and investigate the effects of a novel multifunctional peptide from Lingzhi on the melanogenic enzymes in melanoma cells by a targeted-proteomics approach. The multifunctional peptide was de novo sequenced by LC-MS/MS to be NH_2_-PVRSSNCA-CO_2_H (octapeptide). This sequence was chemically synthesized by solid-phase peptide synthesis (SPPS). The antioxidant ability of the synthesized octapeptide was measured by the DPPH, ABTS, and FRAP assays. The results showed that the peptide exhibited an antioxidant activity equal to 0.121 ± 0.01 mg equivalent to ascorbic acid, 0.173 ± 0.03 mg equivalent to gallic acid, and 2.21 ± 0.23 mM equivalent to FeSO_4_, respectively, which is comparable to these well-known antioxidants. The proteomics approach identified a total of 5804 proteins and several pathways involved in the effects of the octapeptide in melanoma cells. Targeted proteomics revealed three specific proteins associated with pigmentation including Rab29, Dct, and Tyrp1. The Rab29 and Dct were upregulated whereas Tyrp1 was downregulated in the octapeptide treatment group. These findings could be used in the understanding of the molecular functions of the multifunctional octapeptide on melanogenic enzymes, supporting its potential as a therapeutic and cosmetic ingredient.

## 1. Introduction

Lingzhi (*Ganoderma lucidum*) is an oriental fungus known as a traditional Chinese medicine. It exerts several benefits to human health, for example, it reduces oxidative stress, reduces the risk of prostate cancer, and strengthens the immune system [1,2,3]. Lingzhi is an excellent source of bioactive peptides, which are low molecular-weight amino acid sequences with between 3 and 20 residues in length. These peptides were reported to exhibit health-promoting functions [3].

Oxidative stress is a disturbance in the redox homeostasis that occurs by the imbalance between the free radicals (unstable reactive molecules) and antioxidant machinery. The excess free radicals have devastating effects on health, leading to diseases such as cardiovascular disease (CVD), type 2 diabetes (T2D), Alzheimer’s disease, and many types of cancer [4,5]. Well-known chemicals that are used as antioxidants such as butylated hydroxytoluene (BHT), tertiary butyl hydroquinone (Tert-BHQ), and butylated hydroxyanisole (BHA; E-320) have been reported to have some adverse effects on human health and are now restricted in some food products [6]. Hence, the discovery of novel peptide-based antioxidants, which are more biocompatible, would be beneficial for both the human health and food industries.

Tyrosinase is a key regulatory enzyme that is essential for melanin biosynthesis and pigmentation in mammals [7]. Melanin production occurs in melanocytes through a biological cascade with the tyrosinase enzyme catalyzed reaction being the rate-limiting step. The general biological function of melanin is to protect skin injury from exposure to extrinsic stress such as UV radiation, dust, and pollution. However, the overaccumulation of melanin may result in skin-related problems such as melasma, freckles, age spots, and senile lentigo. Thus, the discovery of potent inhibitors of tyrosinase has become increasingly interesting to the therapeutic and cosmetic industries. Although natural and chemical tyrosinase inhibitors such as hydroquinone, 3-O-ethyl ascorbic acid, arbutin, kojic acid, and resveratrol have been identified [8,9], some of these substances (i.e., hydroquinone, kojic acid, and arbutin) exhibit undesirable side effects including dermatitis, cytotoxicity, and potential carcinogenicity [9,10]. Therefore, it is necessary to identify and characterize new tyrosinase inhibitors that are both safe and effective.

In our recently published studies, the biological activities of the Linzhi extracts (hydrolysate) were shown and proven to be excellent for the tyrosinase inhibition activity amongst the others [3,11]. However, the effects of an individual peptide were not studied, and the molecular mechanism was unknown. Therefore, we set out to identify a new bioactive peptide from the Lingzhi hydrolysate by first conducting in vitro tyrosinase activity screening, followed by the identification of the peptide sequence that showed the highest peptide ion intensity. We believe that the highest-intensity peptide ion can reflect the biological effects of a major component in the sample.

Moreover, multifunctional bioactive peptides that can scavenge free radicals and also have tyrosinase inhibitory activity could be a potential active ingredient in cosmetics and in the treatment for hyperpigmentation. Generally, exploring and identifying bioactive peptides in natural products have been conducted via hydrolyzing proteins in the raw materials with proteases [12]. Subsequently, the bioactive peptides that exhibit health-promoting effects can be identified using mass-spectrometry. The identified peptides can be re-synthesized, purified, or modified and developed into a variety of therapeutic agents and commercial food products. However, to develop such a novel substance, establishing the biological mechanisms in cells is necessary. Therefore, a targeted proteomics approach was used to address this knowledge gap by focusing on specific proteins in the melanogenesis pathway. The aims of this study were to identify a new potent and multifunctional peptide and explore the biological mechanisms involved in melanogenesis.

## 2. Results

### 2.1. Isolation and Identification of Multifunctional Octapeptide Derived from Lingzhi Protein Hydrolysate

**Figure 1:**
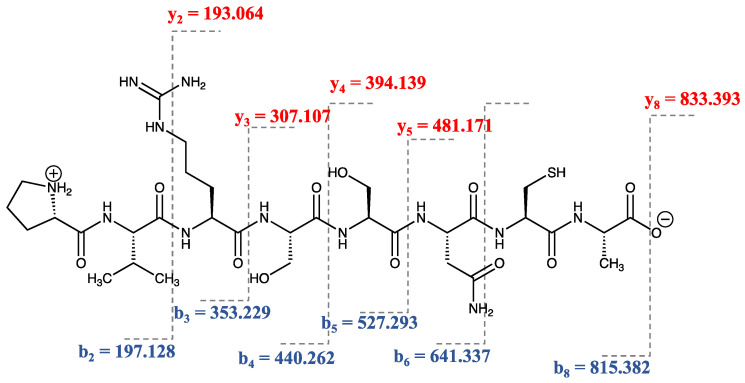
The fragmentation pattern of the highest ion intensity (M + H) + = 832.38; *m*/*z* = +1) from the hydrolysate. The fragment ions generated from the peptide sequence align with the spectrum. N-terminal ions (b-ion series) are shown in blue and C-terminal ions (y-ion series) are shown in red. The chemical structure was generated using the ChemDraw 19.1 academic licensed software.

The initial step to entirely release the protein hydrolysate from Lingzhi was performed in combination with enzymatic treatment. Column-based ultrafiltration is generally used in the partial purification, concentration, and size-fractionation. To fractionate the protein hydrolysate, ultrafiltration with 3 kDa was used to obtain peptides with a smaller than 3 kDa in molecular weight since most bioactive peptides are generally 3–20 amino acid residues [13]. C18-RP separation technique is used to remove insoluble compounds and obtain hydrophilic peptides. After fractionation based on size and hydrophobicity, the water-soluble and smaller molecular-weight peptides proceeded to sequencing by LC-MS/MS. A de novo sequencing algorithm was used to analyze the maximum peptide ion intensity in the hydrolysate. We found that the highest peptide ion (*m*/*z*) was 833.393 with a +1-charge ion. This peptide ion was fragmented by collision-induced dissociation (CID) on the peptide backbone fragmentation pattern to construct MS/MS spectra with b-ion and y-ion intermixed series (Supplementary Figure S1). The sequence of the peptide was constructed as shown in Figure 1 based on the y-ion series including 193.25, [y-NH_3_] 290.14, 394.16, 481.21, 833.39 and the b-ion series including 197.12, 369.14, 527.21, 641.37, 815.32. 

The de novo sequencing of an amino acid sequence was assigned based on the mass difference for a series of successive peptide b-ion and y-ion series for a MS/MS spectrum. The sequence was identified as Proline-Valine-Arginine-Serine-Serine-Asparaine-Cysteine-Alanine (PVRSSNCA), which contains eight amino acid residues. The sequence was then re-synthesized by solid-phase peptide synthesis (SPPS) to obtain a single and highly pure synthetic peptide for further studies and analysis.

### 2.2. Peptide Synthesis by SPPS Approach

The peptide sequence, PVRSSNCA, was chemically synthesized by solid-phase peptide synthesis (SPPS). Two crucial quality control parameters applied in SPPS were peptide purity and mass deviation. A purity and molecular mass analysis of the crude synthetic peptides by analytical HPLC and mass spectrometer is shown in Supplementary Table S1.

The peptide detection by the HPLC chromatogram using maximum absorbance at 220 nm was performed to calculate the peptide purity by calculating the ratio of the peak area of the peptide of interest and the total area for all peaks. These synthesized peptides revealed 89% of purity (Supplementary Figure S2). The mass spectrum of the LC-MS was used to confirm the peptide mass by the calculation of the largest peak area corresponding to the PVRSSNCA peptide. The mass deviation of the synthesized peptide was 0.0396%. With an 89% purity, this indicates that the identity of the synthetic peptide was acceptable to be used for further biochemical assays such as antioxidant assays, enzyme kinetic reactions, and semi-quantitative applications [14]. 

### 2.3. In Vitro Radical Scavenging Assay of the Octapeptide by DPPH, ABTS, and FRAP Assays

Since the antioxidant activity of a substance should be determined by different assays to study the radical scavenging mechanisms, the in vitro radical scavenging potential of the synthesized octapeptide was determined by three different common methods: 2,2-diphenyl-1-picrylhydrazyl (DPPH), 2,2′-azino-bis(3-ethylbenzothiazoline-6-sulfonate) (ABTS), and ferric reducing antioxidant power (FRAP) assays. We compared the antioxidant activity of our newly synthesized peptide to established assays such as the ascorbic acid equivalent antioxidant capacity (AsEAC), gallic acid equivalent antioxidant capacity (GaEAC), and FeSO_4_ molar equivalent reduction capacity by the DPPH, ABTS, and FRAP assays, respectively. These methods were employed as they are commonly used in the screening of antioxidant abilities and primarily characterized the antioxidative compounds [3,15]. The octapeptide at a concentration of 1 mg/mL was used in these assays. The scavenging activities were calculated to be 0.121 ± 0.01 AsEAC and 0.173 ± 0.03 GaEAC for the DPPH and the ABTS assay, respectively. In the FRAP assay, the octapeptide exhibited a powerful reductant ability at 2.21 ± 0.23 mM equivalent to FeSO_4_. These data indicated that the new synthetic peptide showed comparable antioxidant activities. 

### 2.4. In Vitro Tyrosinase Inhibition Assay

**Figure 2:**
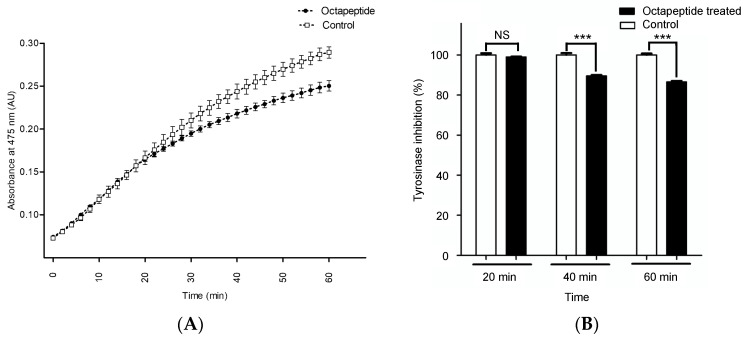
The in vitro tyrosinase activity inhibition. (**A**) The inhibitory effect of 0.05 mg/mL octapeptide on tyrosinase in a time-dependent manner. (•) and (o) represent the octapeptide and control groups, respectively. (**B**) The endpoint inhibition (at 60 min) of the octapeptide on the tyrosinase activity. Clear and solid bars represent the control and octapeptide groups, respectively. Data were represented as mean ± SD from triplicate results. Asterisks (***) indicate the significance levels of *p* < 0.001 and NS, not significant.

To understand how the octapeptide modulates melanogenic enzymes, the activity of tyrosinase in the presence of the peptide was measured by the colorimetric assay. We used the time-dependent assay to confirm that the inhibition reaction occurred with dopachrome accumulation (absorbance at 475 nm). The time-dependent tyrosinase inhibition and the inhibition percentage of the octapeptide at a 50 ug/mL concentration are shown in Figure 2.

The time-dependent inhibition of the octapeptide exhibited a nonlinear rate of inhibition, as shown in Figure 2A. During the first 30 min of the reaction, the inhibitory effect was not observed as the rate was similar to the control. After 32 min, the octapeptide had a distinct inhibitory effect compared to the control. At the end of the monitoring (60 min), the tyrosinase inhibitory effect of the octapeptide was calculated to be 13.48 ± 2.12%, *p*-value ≤ 0.01 (Figure 2B). Although the 60-min reaction could not reach the steady-state of the enzyme kinetics, it was long enough to cover all of the reactions for the discovery of a new tyrosinase inhibitor [16].

### 2.5. Cytotoxicity Assessment and Treatment Condition

**Figure 3:**
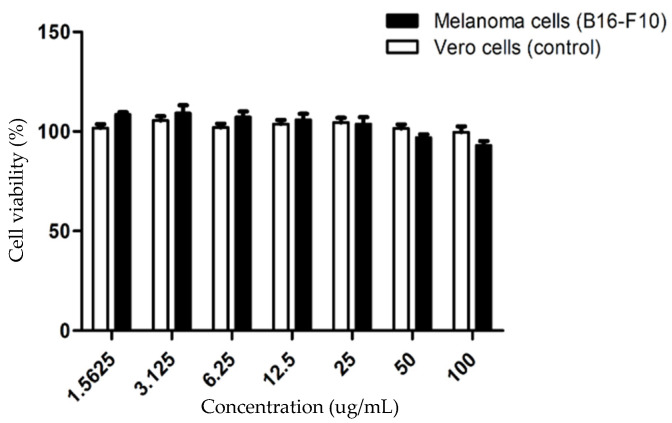
The concentration-dependent effect of the octapeptide on melanoma and Vero cell viability using the MTT assay. Melanoma cells as (■) and Vero cells as (□) were incubated with 1.5625–100 μg/mL octapeptide for 48 h. Data are shown as mean ± SD from triplicate results.

The assessment of cytotoxicity is important in developing the octapeptide toward its practical use in therapeutic and cosmetic products. The cytotoxicity of the octapeptide was investigated in the kidney cell line as normal cells, and the mouse melanoma cells represent melanin producing cells [17,18]. After treatment with the octapeptide for 48 h, the cell cytotoxicity was evaluated by the MTT assay, a colorimetric assay use to assess the cell metabolic activity, reflecting the number of viable cells present, as illustrated in Figure 3. 

The octapeptide at the concentration ranging from 1.5625 μg/mL to 100 μg/mL did not exhibit significant cell death for both the melanoma cells and the Vero cells, which serve as a control normal cell line, indicating the potential application of the octapeptide in therapeutic and cosmetic products. Next, we used the octapeptide at 50 μg/mL, the same as that used in the in vitro tyrosinase inhibitory assay, which also did not significantly affect the melanoma cell viability. Additionally, to confirm that the octapeptide was taken into the cells, we determined the amount of the octapeptide that was left in the medium after 24-h treatment, under the assumption that the remaining peptide in the medium was not absorbed by the cells. We treated cells with 10 μg/mL of the octapeptide and incubated them for 24 h prior to the collection of the medium in triplicate. Using the standard curve of the octapeptide (Supplementary Figure S4) obtained by LC-MS/MS, we found that approximately 2.39 μg (24%) of the octapeptide could be detected in the culture medium (Supplementary Table S2). Therefore, we could presume that the remaining peptide in the culture medium did not pass though the cell membrane. Thus, these data suggest that approximately 76% of the octapeptide could pass through the cell membrane, thereby exhibiting the biological effects.

### 2.6. Targeted-Proteomics Analysis of the Octapeptide in Melanoma Cells

**Figure 4:**
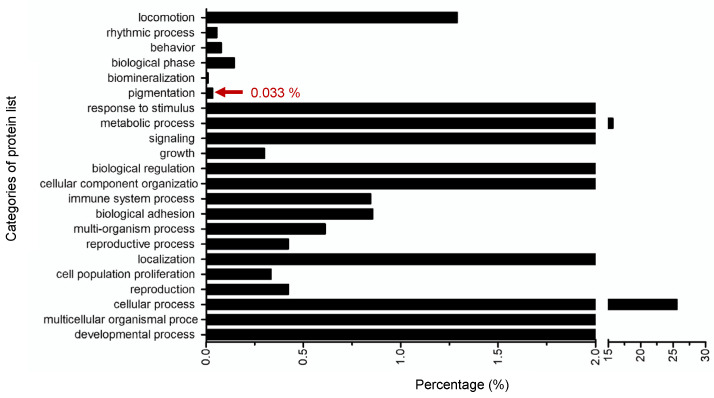
Gene Ontology (GO) enrichment analysis of the proteome dataset represented in the bar chart according to their biological functions. Enrichment analysis classified the protein dataset into 22 groups as the percentage of the total proteome. Three proteins were classified as pigmentation, which was three proteins (0.033%) of the proteome data.

Although using the tyrosinase enzyme extracted from a mushroom for an in vitro tyrosinase inhibitory assay has been widely studied as a routine screening for tyrosinase inhibition [19], tyrosinase from the mushroom is located in the cytoplasm and is different from the mammalian tyrosinase that is found on the membrane of melanocytes [20]. Therefore, cell-based assays are also usually utilized to identify the potential tyrosinase inhibitor. In our study, the effects of the octapeptide at 50 μg/mL in melanoma cells was investigated by a proteomics approach. According to the LC-MS/MS based proteomics results, 5803 proteins have been found to be associated with the effects from the octapeptide (Supplementary Data S1; The mass spectrometry proteomics data have been deposited to the ProteomeXchange Consortium via the PRIDE partner repository with the dataset identifier 606 PXD017234). The results revealed that 686 proteins were differentially expressed in the treated samples compared to the control with the significance level of ANOVA2 < 0.05 between six LC-runs. To classify the overall protein functions in the cellular machinery, the proteome dataset was subjected to Gene Ontology analysis in the category of biological function, as illustrated in Figure 4. 

Based on the physiological processes found in the proteomics result, the protein dataset was classified into 22 functional groups, mainly comprised of the cellular process (25.60%) and metabolic process (15.70%). Remarkably, this classification revealed proteins clustered in pigmentation (Gene Ontology (GO): 0043473), which directly related to the tyrosinase activity. There were three proteins classified in the pigmentation cluster as shown in Table 1, confirming that the octapeptide was indeed involved in the tyrosinase and pigmentation inhibition in a skin-derived cell model.

## 3. Discussion

Our studies used an initial hot water extraction process to release the entrapped protein inside the Lingzhi, followed by enzymatic treatments with pepsin and trypsin, respectively. In previous studies, for the optimization of peptide release from intact Brewers’ spent grain, an initial pre-extraction was required by using alkaline extraction to release the tightly entrapped protein [21]. A strategy to enhance peptide release from the Brewers’ spent grain involves a combination of enzymatic treatment such as carbohydrases (Depol 740 and Econase) and proteases (Alcalase and Promod 439), resulting in the release of proteins. Because the characteristics of Brewers’ spent grain and Lingzhi are the same regarding being a water-insoluble rigid biomass and containing chemical components, proteins are tightly trapped inside the rigid surface structure. Therefore, we used a similar method to obtain the protein hydrolysate from Linzhi [3,22]. We then used LC-MS/MS and de novo sequencing to identify the peptide, which was purified from ultrafiltration to obtain only smaller peptides. This peptide was determined to be NH_2_-PVRSSNCA-COOH, which was then re-synthesized by SPPS and characterized to confirm its bioactivities in the next study, the antioxidant and the tyrosinase inhibition activities. As the net charge of the octapeptide is +1, the octapeptide could act as an electron acceptor to convert free radicals to a stable species and terminate the radical reactivity. This octapeptide includes two hydrophobic amino acid residues, alanine and valine, which may play an important role in scavenging free radicals [23]. The presence of valine at the N-terminus and alanine at the C-terminus may engage with the free radicals at the interface of the water–lipid as lipid bilayer at the biological membrane and therefore scavenge the free radicals [24]. Interestingly, the primary sequence of our novel peptide also contains arginine and valine, which are correlated with a tyrosinase inhibitory screening approach. The SPOT synthesis, generally used to screen peptide–protein interaction, revealed that a potential peptide with tyrosinase inhibitory activity should preferably contain arginine (and/or phenylalanine) in combination with leucine (and/or alanine) and valine. 

**Figure 5:**
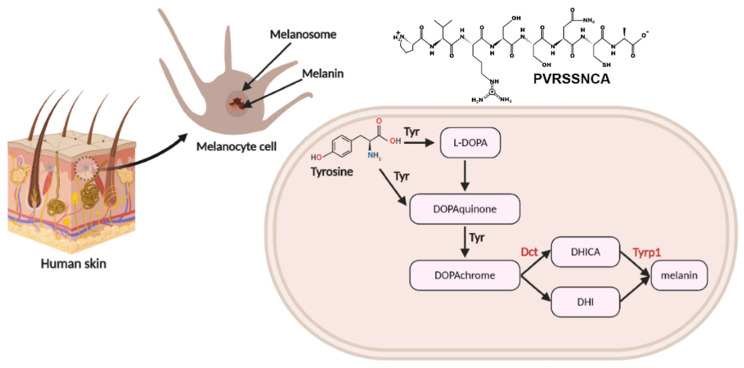
The schematic of the tyrosinase pathway in melanocytes. The diagram shows the enzymatic cascade converting tyrosine to melanin via the tyroninase (Tyr), L-dopachrome tautomerase (Dct), and 5,6-dihydroxyindole-2-carboxylic acid oxidase (Tyrp1) route. Proteomic analysis identified two proteins, Dct and Typ1, highlighted in this diagram. The image was generated with some modifications using the BioRender platform (on the individual license) https://biorender.com (generated on 30 August 2021).

Proteins associated with pigmentation were identified in our targeted-proteomics analysis in melanoma cells, which included Ras-related protein Rab-7L1 (Rab29), L-dopachrome tautomerase (Dct), and 5,6-dihydroxyindole-2-carboxylic acid oxidase (Tyrp1). Rab29 and Dct were upregulated whereas Tyrp1 was downregulated in the octapeptide treatment group (Figure 5). 

**Figure 6:**
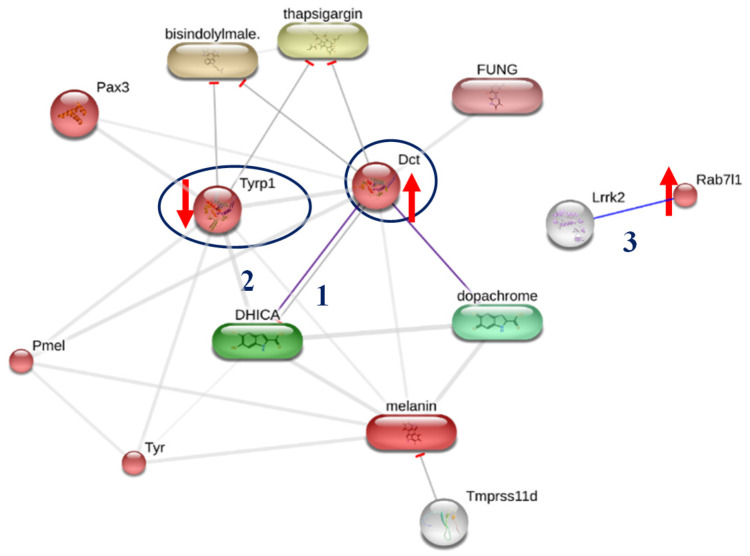
The protein network established by the STITCH protein–ligand interaction (http://stitch.embl.de; accessed on 29 August 2021). Three proteins from the proteomics analysis (Tyrp1, Dct, and RAb7la) were used to construct their interaction. Tyrp1 and Dct are associated with melanin production via DHICA, dopachrome, and Tyr protein. Up-arrow and down-arrow indicated the up- and down-regulation in the octapeptide treatment group.

These proteins are located in the melanosome within melanocytes. Melanin biosynthesis is a complex enzymatic pathway that occurs in melanocytes. The identified proteins, Dct and Tyrp1, are illustrated in a schematic diagram in Figure 5. Although there are many enzymes associated with melanin production, tyrosinase is a major actor in pigment synthesis. Tyrosinase related protein 1 (Typ1) and dopachrome tautomerase (Dct) also have an important function in this process [25]. The STITCH protein–ligand interaction was applied to visualize the ligand–protein interaction network [26]. The association network of Ras-related protein Rab-7L1 (Rab29), L-dopachrome tautomerase (Dct), and 5,6-dihydroxyindole-2-carboxylic acid oxidase (Tyrp1) in the melanin production is shown in Figure 6. 

Melanin biosynthesis (eumelanin and pheomelanin) is a complex pathway enzymatic cascade. The molecular function of Dct is catalyzing the transformation of L-dopachrome into the 5,6- dihydroxyindole-2-carboxylic acid (DHICA) (pathway no. 1). Tyrp1 catalyzes the oxidation of the indolic intermediate 5,6-dihydroxyindole-2-carboxylic acid into 5,6-indolequinone-2-carboxylic acid (pathway no.2). Ras-related protein Rab-7L1 is a key regulator in vesicle trafficking in the organization of the constituents in the assembly or disassembly of a melanosome (pathway no.3). Proteins working in melanin synthesis, tyrosinase (Tyr), Tyrp1, and Dct, are in contact to establish an enzyme complex. Furthermore, the in vitro tyrosinase activity is more stable with the presence of both Tyrp1 and Dct [27]. In addition, the co-expression of Tyr and Tyrp1 or Dct in the melanocytes also increased the melanin content [28]. The unbalance of Dct and Tyrp1 in the octapeptide-treatment group may affect the Dct–Tyrp1 interaction and destabilize the enzyme-complex. This assumption was correlated to the mutant Tyrp1 in murine melanocytes, which showed that the tyrosinase activity decreased quicker than the normal Tyrp1 murine melanocyte cells [29]. To utilize the peptide in cosmeceutical applications, it may require more structural information and characterization in the biological systems. However, our studies may be a good starting point toward the real usage in vivo and in humans.

## 4. Materials and Methods

### 4.1. Purification and Identification of Multifunctional Octapeptide Derived from Lingzhi Protein Hydrolysate

Lingzhi (150 g) was powdered using an ultracentrifugal mill (Retsch Co., Haan, Germany) equipped with a sieve (diameter = 1 mm^3^) at 8000 rounds per minute (Supplementary Figure S1). The Lingzhi protein hydrolysate was initially prepared by hot water-based extraction (at 121 °C for 25 min) followed by enzymatic digestion (0.15 mg pepsin for 12 h and 0.15 mg trypsin for 6 h). The extract was filtered and concentrated in a rotary vacuum evaporator at 50 °C. The solution was freeze-dried and then reconstituted in deionized water. High-molecular weight peptides were removed by a 3 kDa molecular weight cut-off (Vivaspin, GE Healthcare, Chicago, IL, USA). After that, enrichment of the water-soluble peptide from the extraction was performed by a Sep-Pak C-18 (Waters, UK). The protein hydrolysate (100 ng) was protonated by formic acid and subjected to an Impact II UHR-TOF MS System (Bruker Daltonics Ltd., Ettlingen, Germany) coupled to a nanoLC system: UltiMate 3000 LC System (Thermo Fisher Scientific, Waltham, MA, USA). The peptide was fractionated with a linear gradient of 5–60% B in 60 min (flow 0.3 uL/min; solvent A: 0.1% formic acid/water, solvent B: 0.1% formic acid/80% acetonitrile (ACN)/water). Electrospray ionization was carried out at 1.6 kV using the CaptiveSpray. The LC-MS/MS spectra were acquired using data-dependent acquisition (DDA) to automatically switch between the MS and MS/MS acquisition by the auto-MS/MS method. The MS scanning was performed by using a dynamic method with a fixed cycle time of 3 s. Mass spectral information was collected in the +2, +3, and +4 charge state, and *m*/*z* ranged from 400 to 1200. The raw spectra file (.d file) was imported to PeakX studio 10.0 (Bioinformatics Solutions Inc., Waterloo, CA, USA). Automated de novo peptide sequencing of the highest peptide ion intensity was performed with default parameters for the Q-TOF instrument. Briefly, the MS spectra were analyzed with no specific digestion enzyme. The mass error tolerance for MS and MS/MS were 120 ppm and 0.05 Da, respectively. Oxidation (+15.99 Da) on the methionine residue was a variable modification. The CID-Fragmentation series (a, b, c, y, z, b-H_2_O, y-H_2_O, and y-NH_3_), which emphasized the b- and y-ion series, were used to construct the peptide sequence. The acceptable de novo peptide sequences were achieved by filtering the average local confidence (ALC) to ≥80%.

### 4.2. Peptide Synthesis by Solid Phase Peptide Synthesis (SPPS) Approach

The synthesis of the peptide was performed using solid-phase peptide synthesis (SPPS) based on the Fmoc/tBu synthetic amino acids as building blocks followed by the standard protocol [30]. The additional detailed synthetic procedure is described in the Supplementary Materials (page 7). Briefly, the peptides were synthesized stepwise on Wang resins (CAS no. 65307-53-1, catalog no. 855002, 100–200 mesh, Merck-Sigma-Aldrich, St. Louis, MO, USA). The resin was incubated in N-methylpyrrolidinone (NMP) for 16 h. The side-chain amino acid with the N-protection group as building blocks, Fmoc-Ala-OH, Fmoc-Cys(Trt)-OH, Fmoc-Asn-OH, Fmoc-Ser(tBu), Fmoc-Ser(tBu), Fmoc-Arg-OH, Fmoc-Val-OH, and Fmoc-Pro-OH, were mixed with HATU as a coupling reagent for each peptide elongation step. Finally, the peptides were deprotected and cleaved from the resin by TFA/water/TIPS = 93:5:2 (*v*/*v*). The resin was removed by vacuum filtration, the obtained synthetic peptide was precipitated with cold diethyl ether by 1:10 (*v*/*v*), and then pelleted by centrifugation. The crude peptide was reconstituted in 0.1% formic acid/water and subjected to RP-HPLC and LC-MS to evaluate the peptide purity and observed peptide mass. The RP-HPLC analysis was performed on an Inertsil ODS-3 (4.6 mm × 250 mm) analytical column with a linear gradient applied from 5% to 95% solvent B, which was 0.05% trifluoroacetic acid in 100% acetonitrile (*v*/*v*) and solvent A, which was 0.065% trifluoroacetic acid in water (*v*/*v*), with a gradient time of 25 min. The flow rate was 1.0 mL/min at room temperature. The mass measurement error calculation of the synthetic peptides was conducted by the following equation [31].

$$\mathrm{Molecular}\;\mathrm{mass}\;\mathrm{error}\;\left(\%\right)=\frac{\Delta \mathrm{mass}}{\mathrm{Theoretical}\;\mathrm{mass}}\text{×}100$$

where Δmass = observed mass from the LC-MS and the theoretical mass is from the atom composition calculation.

### 4.3. In Vitro Radical Scavenging Assay of the Octapeptide by DPPH, ABTS and Ferric Reducing Antioxidant Power (FRAP) Assay and Tyrosinase Activity Inhibition Assay

For the DPPH scavenging activity, 10 µL of the octapeptide (1 mg/mL) was mixed with 200 µL DPPH solution (0.2 mM in methanol). The control sample composed of 10 μL of methanol and 200 μL of DPPH (0.2 mM). The DPPH scavenging activity of the samples was expressed as the ascorbic acid equivalent antioxidant capacity (AsEAC). For the ABTS scavenging activity, ABTS radical solution (7 mM ABTS stock solution with 2.45 mM potassium persulfate) was diluted in 5 mM phosphate buffer saline (Gibco, Waltham, MA, USA), pH 7.4, to acquire an absorbance of 0.50 at 734 nm before performing the assay. A total of 10 µL of the octapeptide (1 mg/mL) was mixed with 200 µL of the ABTS solution. The reaction was incubated at room temperature in the dark for 10 min. The absorbance of the mixture was evaluated at 734 nm. The ABTS scavenging activity of the samples was expressed as the gallic equivalent antioxidant capacity (GaEAC). For the FRAP scavenging activity, the assay was performed as described from Benzie, I.F. [32] with minor modifications. Briefly, the working solution (10 mM TPTZ/20 mM FeCl_3_ in 300 mM sodium acetate) was freshly prepared and mixed with 10 µL of the octapeptide (1 mg/mL). The reaction was incubated at room temperature in the dark for 30 min. The absorbance was measured at 593 nm. The FRAP scavenging activity of the samples was expressed as FeSO_4_ molar equivalents.

### 4.4. Tyrosinase Inhibition Assay

The inhibitory effect of the octapeptide was determined against mushroom tyrosinase. The mixture of 10 µL of tyrosinase (20 enzyme unit/mL) in 50 mM phosphate buffer, pH 6.8, was added to 96-well plates containing 10 µL of the octapeptide (final concentration = 0.05 mg/mL), then 180 µL of 1 mM L-DOPA was added to start the reaction. The measurement was performed in triplicate (n = 3). The mixture was incubated at 37 °C for 5 min. Monitoring the inhibitory activity by measuring the absorbance at 475 nm for 60 min in a kinetic manner (reading every 1 min) using a microplate reader (Synergy H1, Biotek Inc., Winooski, VT, USA). The tyrosinase inhibitory activity was expressed as a percentage of inhibition at the end-point reaction (t = 60 min) by the equation.

$$\%\mathrm{inhibition}=\frac{\left({\mathrm{Abs}}_{\mathrm{sample}}-{\mathrm{Abs}}_{\mathrm{blank}}\right)}{{\mathrm{Abs}}_{\mathrm{blank}}}\times 100$$

where Abs_sample_ is the absorbance of the reaction with the octapeptide and Abs_blank_ denotes the absorbance of the reaction without the testing sample (a buffer in each assay was added instead of the testing sample).

### 4.5. Cell Culture and Treatment Condition 

The *Mus musculus* skin melanoma cell line (B16-F10 ATCC, CRL-6475) and *Cercopithecus aethiops* kidney normal cell line (Vero ATCC, CCL-81) were used to explore the cell cytotoxicity. The effect of the octapeptide on the skin was evaluated in skin carcinoma cells. The cells were cultured in Dulbecco’s modified Eagle’s medium (DMEM) containing 10% fetal bovine serum (FBS) and 100 IU/mL of penicillin in a humidified atmosphere containing 5% CO_2_ at 37 °C. The cell viability was analyzed using the MTT (3-(4,5-dimethylthiazol-2-yl)-2,5-diphenyltetrazolium bromide) assay. Briefly, the cells were seeded at a density of 2 × 10^4^ cells per well in a 96-well plate. Then, the cells were treated with octapeptide (concentration range = 0.1–0.0016 mg/mL) added into the culture medium. After incubation for 24 h, the cell viability was measured by the optical absorbance at 630 and 570 nm using a microplate reader and transformed into the percentage of cell survival [33]. To study the effect of octapeptide on melanoma, the cells were seeded at a density of 1 × 10^5^ cells/well in a 6-well plate and cultured for 24 h. Then, they were treated with octapeptide at no observed adverse effect level (NOAEL) at 50 μg/mL and further cultured for 48 h. 

### 4.6. Determination of the Peptide Concentration in Culture Medium Using LC-MS/MS 

In a 24-well plate growing the culture of the mouse fibroblast cell line (L929, 20,000 cells/well) for 24 h prior to the treatment with our octapeptide 10 ug/mL) for an additional 24 h, after which the culture medium of the treated and untreated (PBS) samples was collected in triplicate. To quantify the amount of the peptide in the culture medium, we added methanol to the sample at 10:1 ratio (*v*/*v*). The supernatant was subjected to LC-MS/MS (n = 3) to calculate the amount of peptide that remained in the culture medium using the standard curve of the peptide concentration (40, 20, 10, 5, 2.5 ppm or μg/mL) [33]. 

The stock peptide solution (5 mg/mL) was freshly prepared in water (LC-MS grade). Working solutions of these compounds were prepared from the above stock by dilution with 0.1% formic acid/water (*v*/*v*) for the analytical method validation and the standard curve construction. For the analytical method validation and standard curve construction, stock solutions were diluted to obtain the working solutions in the range of 2.5 ppm–40 ppm. The LC-MS/MS analysis was conducted using a Thermo Q-Exactive Quadrupole Orbitrap Mass Spectrometer, coupled to an UltiMate 3000 LC system (HPLC). The analytes were separated using a Hypersil GOLD™ C18 (2.1 mm × 10 mm, 1.9 µm; Thermo Scientific) held at 45 °C. A total of 5 µL of sample injections were used at a flow rate of 0.3 mL/min. The mobile phase was comprised of water with 0.1% formic acid (A), and acetonitrile with 0.1% formic acid (B) (LC-MS grade, Sigma). Gradient starting conditions were 95% A (0.1% FA in water) and 5% B (0.1% FA in ACN). Starting conditions were held for 0.2 min before raising to 80% B over 15 min. The column was flushed with 95% B for 2 min before returning to the starting conditions. The total time for each analysis was 20 min. The MS was operated in positive mode. The LC-MS was acquired in parallel-reaction monitoring (PRM) used with the following settings for a resolution at 60,000; AGC target 3 × 10^6^; max IT 250 ms. The samples were injected (n = 3) with the above-mentioned parameter in the LC-MS/MS. The standard curve of the peptide was constructed, and the nominal known concentrations were plotted against the corresponding peak areas using Xcalibur 2.0. 

Following the protocol similar to that in [33], the limit of quantitation (LOQ) and linearity were determined in our experiment. The LOQ of the analytical method, calculated as the concentration of the injected sample to yield a signal-to-noise ratio of ten, was 2.461 ng/mL. The calibration curve of the peptide concentration was established by weighted (w = 1/x) linear regression analysis. Supplementary Figure S4 showed the linearity (R2 = 0.9862) in the range of 2.5–40 ppm or μg/mL, which we used in this experiment. The linear regression equation is shown in Supplementary Figure S4, where X is the concentration of the octapeptide (μg/mL) and Y is the peak area. The LOQ of this quantification method was validated and calculated using the Thermo Xcalibur QuanBrowser 4.2 software [34]. 

### 4.7. Targeted-Proteome Quantitation and Data Analysis

The melanoma cells were disrupted by lysis buffer cocktail (0.5% Triton X-100, 10 mM DTT in 20 mM HEPES-NaOH, pH 8.0) supplemented with protease inhibitor cocktail (2 mM PMSF, 0.3 μM Aprotinin, 0.3 μM, 2 mM EDTA, and 10 μM E-64). The supernatant was collected by centrifugation and subsequently precipitated by ice-cold acetone (1:5 (*v*/*v*)). After precipitation, the protein pellet was reconstituted in 0.2% RapidGest SF (Waters, Wilmslow, UK) in 10 mM Ammonium bicarbonate, then, 50 µg of protein were subjected to gel-free based digestion. Briefly, the protein solution reduced the sulfhydryl bonds by 5 mM DTT at 72 °C for 1 h and alkylated the sulfhydryl groups by 20 mM IAA at 25 °C for 30 min in the dark. Proteolytic digestion was performed by using 500 ng of sequencing grade trypsin (Promega, Ettlingen, Germany) at 37 °C for 3 h. The digested peptides were cleaned-up by C18-ZipTip, dried, and resolubilized in 0.1% formic acid. Protonated peptide solutions were analyzed using the Impact II UHR-TOF MS System (Bruker Daltonics Ltd., Ettlingen, Germany) coupled to a nanoLC system: UltiMate 3000 LC System (Thermo Fisher Scientific, Waltham, MA, USA) equipped with a Nano captive spray ion source. The mass spectrometry was operated in positive ion mode over the range (*m*/*z*) = 150–2200 (Compass 1.9 for otofSeries software, Bruker Daltonics). The mass accuracy (TOF detector) calibrated with the LC/MS tuning mix for ESI (Agilent, Santa Clara, CA, USA) was within 1.6 ppm. The raw LC-MS runs were carried out using CompassXport Version 3.0.9.2 (Bruker Daltonics GmbH, Ettlingen, Germany) to convert the raw spectra (.d file format) to the mzXML data format. 

The mzXML data were evaluated by the relative comparison of the peak intensities of each peptide ion in all datasets by DeCyder MS Differential Analysis software version 2.0 (GE Healthcare Co., Boston, MA, USA) [35]. Peak detection (signal/noise ratio < 2) and peak matching (peptide ion peak alignment) were carried out by the Batch and Pepmatch modules in the DeCyder MS Differential Analysis software. All datasets of the MS/MS peak lists from the Pepmatch module in the DeCyder MS Differential Analysis software were exported to the Mascot generic format (.mgf file format) and submitted for an in-house database search using the Mascot software (Matrix Science, London, UK). The dataset of the MS/MS peak lists was searched against the *Mus Musculus* (Uniprot database, 17027 sequences) for protein identification by the following parameters: trypsin as a digesting enzyme; maximum of three missed cleavages; MS peptide tolerance of 1.0 Da; the MS/MS peptide tolerance of 0.5 Da; the carbamidomethylation modification of the cysteine residue as a fixed modification; and the oxidation of methionine and the acetylation of the protein N-terminus as variable modifications. Significant peptides with an ANOVA lower than 0.05 were accepted. Data normalization for each LC-run was conducted by internal normalization with two peptides from the beta-tubulin protein, which is a housekeeping protein. The mass spectrometry proteomics data were deposited at the ProteomeXchange Consortium via the PRIDE partner repository with the dataset identifier PXD017234. Gene Ontology enrichment analysis by the biological process was performed using the Panther Classification System (http://www.pantherdb.org; accessed on 7 December 2021) [36].

### 4.8. Statistical Analysis

All of the experiments were carried out with at least three independent replicates (n = 3), and all data were expressed as the means ± standard deviation. Analysis of variance (ANOVA) was performed by the DeCyderMS program in the PepMatch modules. The significance in differences was determined by the Duncan’s multiple range test (*p* < 0.05). Determination of the peptide concentration in the culture medium using the LC-MS/MS calibration curve for peptide quantification in the culture medium was calculated using a weighted (1/x) least square regression and the linearity of the calibration curve was determined by the correlation coefficients (R^2^).

## 5. Conclusions

We successfully identified a novel octapeptide from the Lingzhi extract. Our findings suggest that the octapeptide (PVRSSNCA) is multifunctional in terms of being a potent antioxidant and tyrosinase inhibitor. It strongly scavenged free radicals, inhibited the tyrosinase, affected multiple melanogenic enzymes, and showed no significant cytotoxicity in the cells. Targeted proteomics analysis revealed the effects of octapeptide on the proteins in pigmentation associated with tyrosinase and melanogenesis. We strongly believe that this octapeptide may be a significant bioactive agent with the potential for the treatment of skin hyperpigmentation in therapeutic and cosmetic products in the near future.

## Figures and Tables

**Table 1:** The list of identified proteins in pigmentation (Gene Ontology; (GO): 0043473) that are directly associated with tyrosinase.

Protein ID	Protein Name	PANTHER Family/Subfamily	PANTHER Protein Class	Relative Expression (Fold)
Q91YQ1	Ras-related protein Rab-7L1; Rab29	PTHR24073:SF315	-	2.0646
P29812	L-dopachrome tautomerase; Dct	PTHR11474:SF4	oxygenase	2.0460
P07147	5,6-dihydroxyindole-2-carboxylic acid oxidase; Tyrp1	PTHR11474:SF3	oxygenase	0.5034

## Data Availability

Data is contained within the article and supplementary files.

## Supplementary Materials

The following supporting information can be downloaded at: https://www.mdpi.com/article/10.3390/ph15060684/s1, Figure S1: Fragmentation spectra by collision-induced dissociation (CID) on the peptide ion; Figure S2: Crude synthetic peptide chromatographic profile by RP (C18)-HPLC; Figure S3: Mass spectrum of the synthesized octapeptide; Figure S4: The standard curve of the peptide PVRSSNCA concentration; Table S1: Purity and molecular mass information of synthesized peptides from SPPS method; Table S2: Calculated peptide PVRSSNCA content in culture medium from the standard curve; Figure S5: A photograph of the raw material of Lingzhi prior to enzymatic digestion; The detailed solid-phase peptide synthesis procedure.
