# Supplementary material for: Discovery of a Multifunctional Octapeptide from Lingzhi with Antioxidant and Tyrosinase Inhibitory Activity
Source: Pharmaceuticals (Basel). 2022 May 30;15(6):684. doi: 10.3390/ph15060684 (PMC9230030; doi:10.3390/ph15060684)
Supplement: Supplementary file 1 [file pharmaceuticals-15-00684-s001.zip › pharmaceuticals-1660514-supplementary.pdf]

Supplementary material for the manuscript:

Discovery of a multifunctional octapeptide from Lingzhi with antioxidant and tyrosinase inhibitory activity

Yodying Yingchutrakul [1,2†], Sucheewin Krobthong [2,3†], Phakorn Papan[4], Pawitrabhorn Samutrtai[5], Thanisorn Mahatnirunkul[6], Thitikorn Chomtong[6], Nitipol Srimongkolpithak[7], Kiattawee Choowongkomon[3], Theeranuch Jaroenchuensiri[8], Chanat Aonbangkhen[8*]

[1]National Omics Center, National Science and Technology Development Agency (NSTDA), Pathum Thani, 12120, Thailand

[2]Center for Neuroscience, Faculty of Science, Mahidol University, Bangkok, 10400, Thailand

[3]Department of Biochemistry, Faculty of Science, Kasetsart University, Bangkok, 10900, Thailand

[4]Department of Chemistry, Faculty of Science, Chiang Mai University, Chiang Mai 50200, Thailand

[5]Department of Pharmaceutical Sciences, Faculty of Pharmacy, Chiang Mai University, Chiang Mai, 50200, Thailand

[6]National Nanotechnology Center, NSTDA, Pathum Thani, 12120, Thailand

[7]National Center for Genetic Engineering and Biotechnology (BIOTEC), National Science and Technology Development Agency (NSTDA), Pathum Thani, Thailand.

[8]Center of Excellence in Natural Products Chemistry (CENP), Department of Chemistry Faculty of Science, Chulalongkorn University, Bangkok 10330, Thailand

† These authors contributed equally

* Correspondence: C.A., chanat.a@chula.ac.th

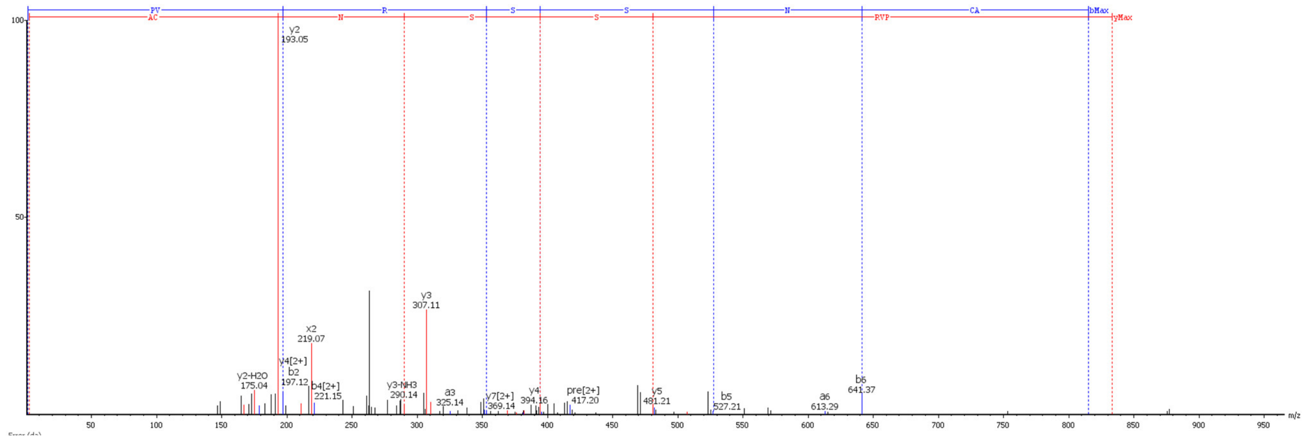

Supplementary Figure S1: Fragmentation spectra by collision-induced dissociation (CID) on the peptide ion (*m/z*) was 833.393 with a +1 charge ion. The sequence of the peptide was constructed based on y-ion series (Red) and b-ion series (blue). The sequence of the peptide is determined by the mass difference between these ion series by de novo algorithm from PeakX studio 10.0 software (Ref: Ma *et al*., *Rapid Commun Mass Spectrom 17*(20), 2337-2342 (2003). The sequence of this peptide ion was Proline-Valine-Arginine-Serine-Serine-Asparaine-Cysteine-Alanine.

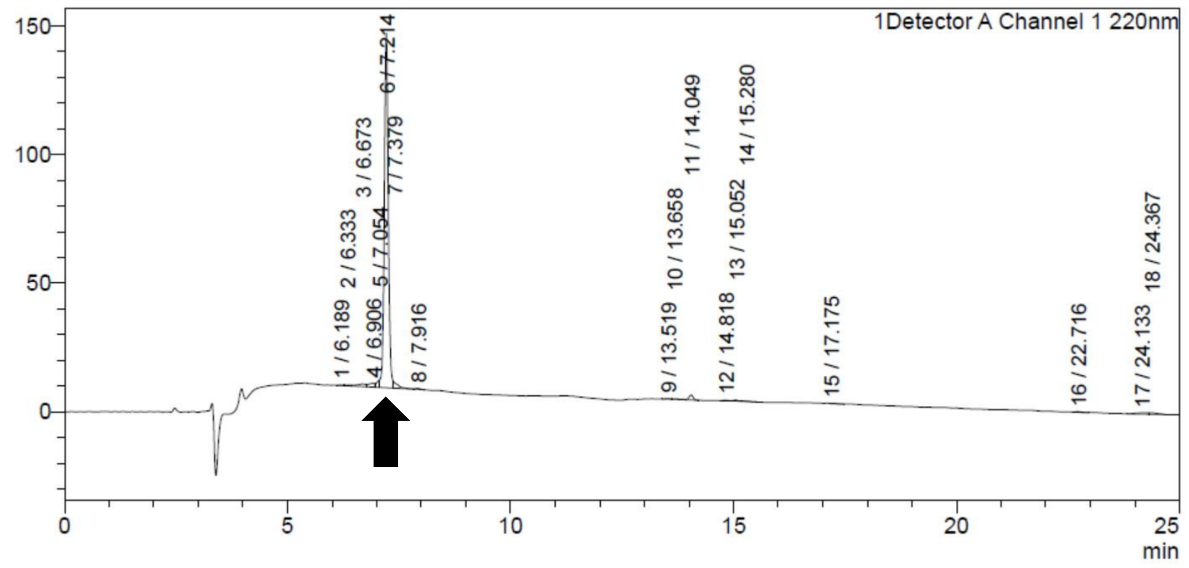

Supplementary Figure S2: Crude synthetic peptide chromatographic profile by RP (C18)-HPLC. The peptide purity was determined by HPLC analysis (peak area), which was monitored at 220 nm as a common wavelength for peptide detection. The synthetic peptide (PVRSSNCA) was eluted at R_t =7.214 minutes with a peak area = 855895, the black arrow indicated the synthetic peptide peak.

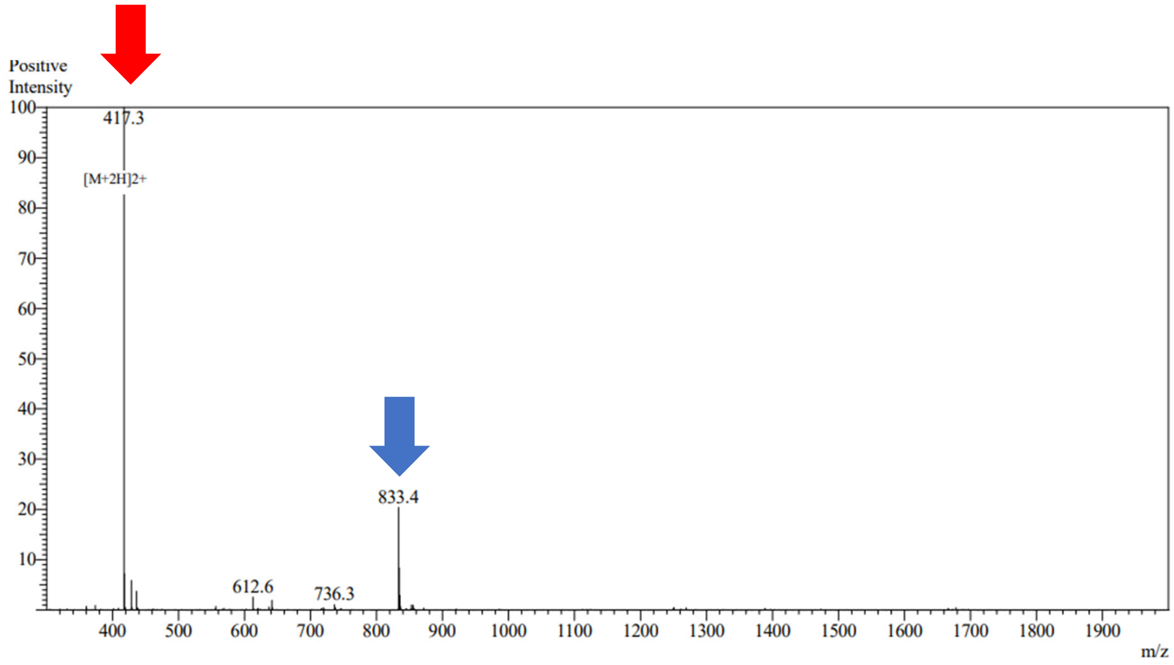

Supplementary Figure S3: Mass spectrum of the synthesized octapeptide. The spectrum shows the mass of $[M+2H]^{2+}$ and $[M+H]^{+}$ of the synthesized peptide ion at 417.3 (red arrow) and 833.4 (blue arrow), respectively. The spectrum confirms that the peptide was successfully synthesized with the free N- and C-terminus.

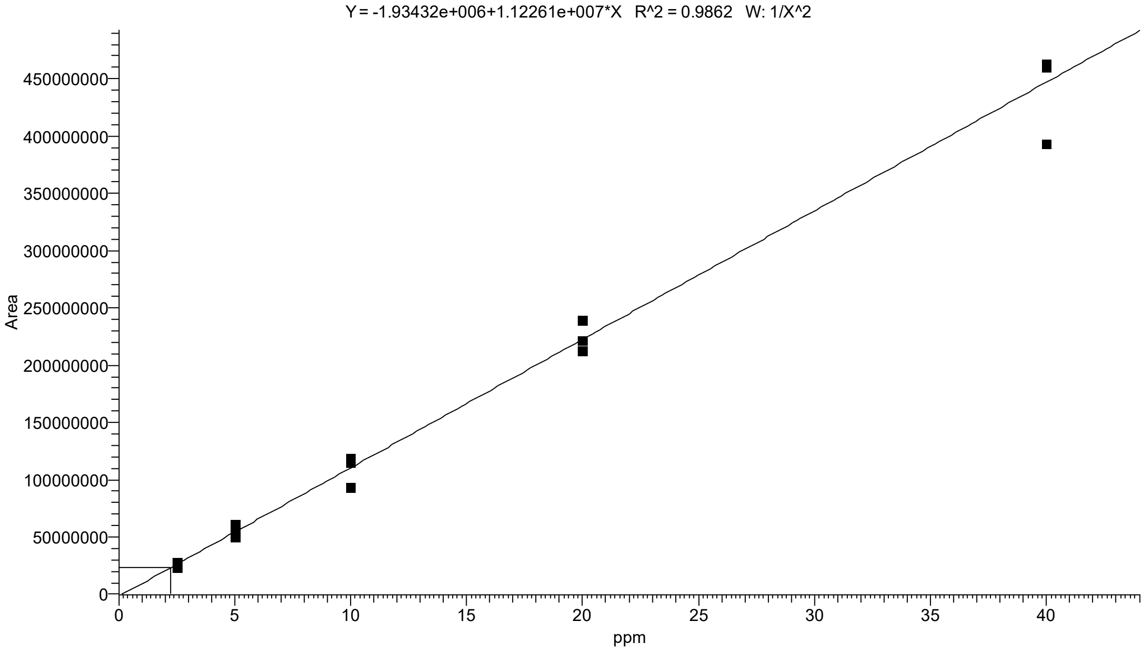

Supplementary Figure S4 | The standard curve of the peptide PVRSSNCA concentration.

Supplementary Table S1. Purity and molecular mass information of synthesized peptides from SPPS method.

**Sequence**	**Theo. Mass (Da)**	**Obser. Mass (Da)**	**Mass Devi. (%)**	**Purity (%)**
PVRSSNCA	832.93	832.60	0.0396	89.0%

Supplementary Table S2 | Calculated peptide PVRSSNCA content in culture medium from the standard curve.

Sample	Calculated amount (μg)	Average calculated amount (μg)	S.D.
Replicate 1	2.3412	2.388	0.034
Replicate 2	2.4237		
Replicate 3	2.3991		

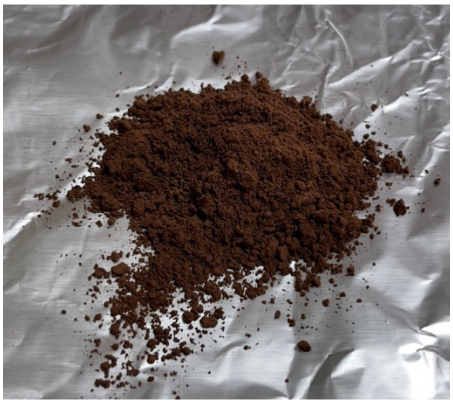

Supplementary Figure S5 | A photograph of the raw material of Lingzhi prior to enzymatic digestion.

The detailed solid-phase peptide synthesis procedure

The SPPS was fully described here; The resin for peptide synthesis (1 g, 1.2 mmol/g) was washed and incubated in roughly 10 mL of N-methylpyrrolidinone (NMP) for 16 hours prior to each reaction. The deprotection solution (50% piperidine in NMP; 4 mL of NMP and 4 mL of piperidine) were added to the resin for 10 minutes with nitrogen gas. The solution was filtered from the resin and the resin was washed using dichloromethane (DCM) and NMP. This process was repeated for a total of four times to prepare the resin for amino acid elongation processes. The side-chain amino acid with the N-protection group as building blocks, Fmoc-Ala-OH, Fmoc-Cys(Trt)-OH, Fmoc-Asn-OH, Fmoc-Ser(tBu), Fmoc-Ser(tBu), Fmoc-Arg-OH, Fmoc-Val-OH, and Fmoc-Pro-OH, were mixed with HATU as coupling reagent for each peptide elongation step. For the elongation processes contain 8 reactions as the followings.

1.1 To obtain resin linked with Alanine (Ala), Fmoc-alanine-OH (3 eq.) was added diisopropylethylamine (DIPEA) (6 eq.) and NMP (8 mL), and the solution was incubated for 30 seconds. The solution was then added to the resin and the mixture was sparged for 20 minutes with nitrogen gas. The solution was filtered from the resin and washed using NMP and DCM. The deprotecting solution 6 mL of 50% piperidine in NMP and piperidine were added to the resin, and the mixture was mixing for 3 minutes. The solution was filtered from the

conjugated resin and was washed using NMP and DCM, and the reaction was analyzed for completion reaction using the Kaiser test before cysteine (Cyt) elongation.

1.2 To obtain resin-Ala linked with Cyt, Fmoc-Cyt-OH (3 eq.) was added DIPEA (6 eq.) and NMP (8 mL), and the solution was incubated for 30 seconds. The solution was then added to the resin-Ala and the mixture was sparged for 20 minutes with nitrogen gas. The solution was filtered from the conjugated resin and washed using NMP and DCM. The deprotecting solution 6 mL of 50% piperidine in NMP and piperidine were added to the resin-Ala, and the mixture was mixing for 2 minutes. The solution was filtered from the conjugated resin and was washed using NMP and DCM. The reaction was analyzed for completion reaction using the Kaiser test before glutamine (Gln) elongation.

1.3 To obtain resin-Ala-Cyt linked with Gln, Fmoc-Gln-OH (3 eq.) was added DIPEA (6 eq.) and NMP (8 mL), and the solution was incubated for 30 seconds. The solution was then added to the resin-Ala-Cyt and the mixture was sparged for 20 minutes with nitrogen gas. The solution was filtered from the conjugated resin and washed using NMP and DCM. The deprotecting solution, 6 mL of 50% piperidine in NMP and piperidine were added to the conjugated resin, and the mixture was mixing for 2 minutes. The solution was filtered from the conjugated resin and was washed using NMP and DCM. The reaction was analyzed for completion reaction using the Kaiser test before serine (Ser) elongation.

1.4 To obtain resin-Ala-Cyt-Gln linked with Ser, Fmoc-Ser-OH (3 eq.) was added DIPEA (6 eq.) and NMP (8 mL), and the solution was incubated for 30 seconds. The solution was then added to the resin-Ala-Cyt-Gln and the mixture was sparged for 20 minutes with nitrogen gas. The solution was filtered from the conjugated resin and washed using NMP and DCM. The deprotecting solution, 6 mL of 50% piperidine in NMP and piperidine were added to the resin-QAQ, and the mixture was mixing for 2 minutes. The solution was filtered from the conjugated resin and was washed using NMP and DCM. The reaction was analyzed for completion reaction using the Kaiser test before Ser elongation.

1.5 To obtain resin-Ala-Cyt-Gln-Ser linked with Ser, Fmoc-Ser-OH (3 eq.) was added DIPEA (6 eq.) and NMP (8 mL), and the solution was incubated for 30 seconds. The solution was then added to the resin-Ala-Cyt-Gln-Ser and the mixture was sparged for 30 minutes with nitrogen gas. The solution was filtered from the conjugated resin and washed using NMP and DCM. The deprotecting solution, 6 mL of 50% piperidine in NMP and piperidine were added to the conjugated resin, and the mixture was mixing for 2 minutes. The solution was filtered

from the conjugated resin and was washed using NMP and DCM. The reaction was analyzed for completion reaction using the Kaiser test before arginine (Arg) elongation.

1.6 To obtain resin-Ala-Cyt-Gln-Ser-Ser linked with Arg, Fmoc-Arg-OH (3 eq.) was added DIPEA (6 eq.) and NMP (8 mL), and the solution was incubated for 30 seconds. The solution was then added to the resin-Ala-Cyt-Gln-Ser and the mixture was sparged for 30 minutes with nitrogen gas. The solution was filtered from the conjugated resin and washed using NMP and DCM. The deprotecting solution, 6 mL of 50% piperidine in NMP and piperidine were added to the conjugated resin, and the mixture was mixing for 2 minutes. The solution was filtered from the conjugated resin and was washed using NMP and DCM. The reaction was analyzed for completion reaction using the Kaiser test before valine (Val) elongation.

1.7 To obtain resin-Ala-Cyt-Gln-Ser-Ser-Arg linked with Val, Fmoc-Val-OH (3 eq.) was added DIPEA (6 eq.) and NMP (8 mL), and the solution was incubated for 30 seconds. The solution was then added to the resin-Ala-Cyt-Ser-Ser-Arg and the mixture was sparged for 30 minutes with nitrogen gas. The solution was filtered from the conjugated resin and washed using NMP and DCM. The deprotecting solution, 6 mL of 50% piperidine in NMP and piperidine were added to the conjugated resin, and the mixture was mixing for 2 minutes. The solution was filtered from the conjugated resin and was washed using NMP and DCM. The reaction was analyzed for completion reaction using the Kaiser test before proline (Pro) elongation.

1.8 To obtain resin-Ala-Cys-Gln-Ser-Ser-Arg-Val linked with Pro, Fmoc-Pro-OH (3 eq.) was added DIPEA (6 eq.) and NMP (8 mL), and the solution was incubated for 30 seconds. The solution was then added to the resin-Ala-Cys-Gln-Ser-Ser-Arg-Val and the mixture was sparged for 50 minutes with nitrogen gas. The solution was filtered from the conjugated resin and washed using NMP and DCM. The deprotecting solution, 6 mL of 50% piperidine in NMP and piperidine were added to the conjugated resin, and the mixture was mixing for 2 minutes. The solution was filtered from the conjugated resin and was washed using NMP and DCM. The reaction was analyzed for completion reaction using the Kaiser test before cleavage synthesized peptides from the resin.

Final synthetic peptides were deprotected and cleaved from the resin by cleavage cocktail containing trifluoroacetic acid (TFA)/water/ triisopropylsilane (TIPS)/ = 93:5: 2 at room temperature. The synthetic peptide was precipitated with cold diethyl ether by 1:10 (v/v)

and collected the crude synthetic peptide pelleted by centrifugation at 10000g, 4°C for 30 minutes. The synthesized peptide product was cleaved from resin as above in order to verify identity using LC-MS. The oveall yield was ~30%. The mass spectrum of the synthesized peptide was shown below. The total mass of the peptide was confirmed by LC-MS. The N- and C-terminus of the peptide were determined to be the free-NH_2 and free-COOH, which matches the peptide detected from the hydrolysates.
